# Memoir on the Operation of Lithotomy

**Published:** 1841-07

**Authors:** 


### Art. XV.

*Mémoire sur l'Opération de la Taille.* Par M. Souberbielle. (*Mémoires de l'Académie Royale de Médecine.* Tome Huitième.)—*Paris*, 1840. 4to, pp. 50.

*Memoir on the Operation of Lithotomy.* By M. Souberbielle. *From the Memoirs of the Royal Academy of Medicine.* Vol. VIII.—*Paris*, 1840.

This paper includes the cases of 50 patients operated upon by M. Souberbielle between the years 1828 and 1834, in 39 of whom the calculus was extracted above the pubes. The high operation for stone has at different periods been revived and practised by some eminent surgeons of our own country, and as often has it again fallen into disuse; it has likewise from time to time obtained its advocates abroad, who have suggested and adopted it as a judicious method of removing calculi in certain cases where the lateral operation presented difficulties and would necessarily be attended with hazard and danger. The subject must, therefore, always be one of considerable interest to the practical surgeon whose experience has taught him to prognosticate and appreciate the difficulties and untoward results incident to what are generally called unfavorable cases, cases which the surgeon would gladly avoid in practice, and which he is only induced to undertake from a sense of duty at the earnest solicitations of his patients. As the experience of M. Souberbielle in the performance of the high operation has doubtless been greater than that of any other living surgeon, and as his practical acquaintance with the perineal section must have enabled him to compare the two methods with sufficient accuracy, we shall owe no apology to our readers for laying before them the result of his practice, together with the reasons which he gives for his preference to the mode of incising the bladder above the pubes. We shall therefore quote the author's own words from the essay appended to the history of the 50 cases, the details of which form the bulk of his communication.

We may first observe that M. Souberbielle opens the bladder by means of the *sonde à dard*, introduced by the urethra and brought out through the linea alba above the pubes. In three cases, where the size of the stone and the close application of the bladder to its surface did not allow the introduction of this instrument, the incision was effected from without with a bistoury which was carried down directly on to the stone. He considers, however, that the *sonde à dard* is by far the best instrument for opening the bladder with precision and accuracy, especially in fat persons and where the bladder is contracted and deeply situated. A syphon catheter is introduced by the urethra immediately after the operation and retained until the wound is healed, generally from fifteen to twenty days. Before removing the catheter he generally tests the soundness of the cicatrice by closing the orifice of the instrument for three or four hours, and allowing the urine to accumulate. This is repeated for a few days. Sometimes the catheter becomes a source of irritation to the patient, or the contractions of the bladder prevent its retention within the viscus. It may in such cases be removed, and the water then escapes either partially or wholly by the wound; but the removal of the catheter does not give rise to any other inconvenience than that of somewhat retarding the recovery of the patient.

"In children," says M. Souberbielle, "although the situation of the bladder offers nothing unfavorable to the high operation, I yet prefer the lateral section, merely because they are too intractable to be properly secured, and the violent struggles which they make, and which can neither be guarded against nor prevented, expose the peritoneum to the danger of being wounded: again, the care and attention required to maintain a catheter in the urethra tends much to irritate and excite a young patient, since every time he is approached he imagines that the operation is about to be recommenced. With certain exceptions, therefore, depending on age and peculiar circumstances, I practise the high operation; and my preference for it is founded on reason and experience. Indeed by this method calculi may always be extracted without reference to their size, their number, their situation, the condition of the prostate, &c.; the operation is less painful than any other mode of cutting into the bladder, and exposes the patient to the fewest casualties. The skin, the linea alba, the cellular tissue, and the bladder are the only textures implicated in the section; whereas in the perineum we encounter a variety of structures which it is difficult to avoid, and the necessary lesion of which may give rise to hemorrhage, to stercoraceous fistulæ, to infiltration, to impotence, &c; none of these dangers are incurred by cutting into the hypogastrium. The history of the post-mortem examinations of those who have sunk after the high operation goes to prove that the operation is never itself a cause of death, but that the fatal event has been the result of previous organic alteration or disposition to disease, whose origin was clearly not referrible to the operation, which indeed would exercise no other influence upon it than that of hastening its development. On the other hand, hemorrhages, infiltration of urine, wounds of the rectum, laceration of the cellular tissue, are the consequence of casualties to which the perineal operation is liable, and which are frequently attended with a fatal result. It is my conviction that the high operation as practised by myself is not obnoxious to these fatal consequences. ........

"As regards the size of calculi, the cases 8, 20, and 48* describe stones of large dimensions which offered no difficulty in their passage through the wound, but whose extraction through the perineum would have been either very difficult, as in cases 8 and 20, or altogether impossible, as in case 48.†

"The facility afforded to the extraction of the stone arises from the circumstance that the incision may be enlarged with impunity to any necessary extent, as was proved in the operation I performed on M. Delaborde, member of the ancient academy of surgery, from whom, at the age of eighty-two, I removed a calculus weighing half a pound.‡

* Case 8. M. Gravet, æt. seventy. The stone was of an ovoid form with a rough surface, and weighed four ounces six drachms. The cure was complete. Case 20. M. Constant, æt. sixty-five. A very hard stone of a flattened ovoid form, measuring one inch and a half in its small, two inches and a half in its long, diameter, and seven inches and a half in its circumference, weighing five ounces one drachm. Around it, near its centre, was a groove which corresponded to a sort of collar formed by the bladder, by which it was encircled and so firmly retained, that the knife was had recourse to before the extraction could be effected. The patient was well by the eighteenth day.

† M. Levesque, æt. sixty-five. Two stones of nearly equal volume, of an ovoid form flattened laterally, weighing together seven ounces two drachms and a half. These calculi were moulded to each other and occupied the entire cavity of the bladder; it was necessary to detach the walls of the viscus from the surface before the forceps could be introduced. The incision was carried down directly into the calculi as the sonde à dard could not be made use of. The patient recovered.

‡ No mention is made as to whether the patient survived the operation.

"As regards the presence of a numerous collection of stones in the bladder, or what is equivalent, a single stone which breaks into fragments; several of the 40 cases are very conclusive evidence in favour of the high operation, but none perhaps so strong as case 1, where, notwithstanding the peculiar anatomical condition of the bladder and the numerous sacculi and cells which it contained, nearly 300 calculi were extracted without leaving one behind.*

"The cases 6, 12, 20, 39, 43, 48, present remarkable and decisive examples of the capability afforded for the extraction of stones by the high operation which it would have been impossible to lay hold of through a perineal opening, because they were retained in pouches or else firmly grasped by the bladder itself. In case 43 the stone, which was situated in a pouch at the fundus of the bladder, could not have been reached by instruments introduced through the perineum, and the manœuvre by which it was finally dislodged from its situation could not have been carried into effect.

"The attachment of calculi to the internal surface of the bladder is not so rare a circumstance as might be imagined, since we find that in 16 out of the 50 cases the stones were either embraced by the bladder or retained in the base of the viscus or else inclosed in pouches, which latter it was in some instances necessary to cut before the dislodgment could be effected.

"Lastly, case 35, in which a considerable deformity of the pelvis would have rendered the extraction of the stone through the perineum impossible while it interfered but slightly with the high operation, is a remarkable instance of the advantage of the latter method under certain rare conditions." (Memoire, pp 87-90.)

M. Souberbielle next proceeds to refute the objections which have been advanced against the high operation. These objections he enumerates as urinary infiltration, hemorrhage, and wounds of the peritoneum; the first two he maintains are much more likely to occur after the perineal section, while the latter he considers as an accident not liable to be followed by mischievous results. It would appear that infiltration of urine into the pelvic cellular tissue (which he remarks as being a common occurrence after the lower operation), only took place once out of his 39 cases. It was however undoubtedly the cause of the patient's death on the fifth day. Infiltration, he says, may always be avoided by making a clean incision into the bladder and taking care not to disturb or tear through the loose cellular tissue connecting the anterior surface of the viscus with the abdominal walls. Hemorrhage, which he also cites as a frequent cause of death after the ordinary operations of stone, occurred but in one case, and, notwithstanding all the efforts to arrest the flow of blood, continued until the patient sank twenty-four hours after the operation. In this instance, remarks M. Souberbielle, the fatal event depended on the peculiar idiosyncracy of the individual. Lesions of the peritoneum, he further remarks, are not likely to occur if proper precaution be used in incising the bladder; and when such an accident does take place, it probably depends on some peculiar condition or alteration of the structures concerned in the operation. In two of the cases which form the subject of this memoir the cavity of the peritoneum was opened. One of the patients died, but in the opinion of our author his death was in nowise to be ascribed to the injury inflicted on the serous membrane, as no traces of peritonitis were found on the post-mortem inspection.

* This patient died on the seventh day after the operation from serous effusion into the cavity of the tunica arachnoidea.

We have now laid before our readers the pith and substance of M. Souberbielle's memoir, and extracted in a condensed form nearly all which can be useful or interesting to the surgeon. A few remaining remarks contained in the text we shall also have occasion to refer to, in the observations with which we purpose to conclude this article; for as, after an attentive perusal of the paper, we feel by no means induced to alter materially our previous views of the subject, or subscribe to our author's prepossession in favour of the high operation, it will be necessary to analyze and test the statements he has put forth with so much confidence, and ascertain how far they are founded on accuracy of fact and soundness of judgment. We are far from wishing to impugn the skill or under rate the labour and experience of an eminent man, but we feel ourselves justified in saying that our author's partiality for a favorite operation, which he may be said to have made his own, has evidently led him into great mistakes, into no slight perversion of facts, and erroneous conclusions; that the effects of the strong bias he has conceived has induced him, in the first place, to depreciate the success and magnify the difficulties of the lateral section in the practice of his professional brethren; while, again, it has caused him to exalt unduly the advantages and merits of the high operation.

In the early part of his memoir, M. Souberbielle quotes the published statements of M. Civiale in relation to the number of deaths which occurred in the Paris hospitals after the lateral operation of lithotomy; and although he throws discredit on their accuracy, perhaps not without justice, he yet makes use of them as subservient to his own views and persuasions.

"If," he says, "we are to give credit to M. Civiale, 35 patients died out of 61 operated upon in La Charité; out of 96 patients cut at the Hôtel Dieu there were 27 fatal results; at Beaujon, 6 died out of 11; in the Hôpital Necker, 11 out of 14. I am far from pledging myself to the assertions of M. Civiale but I certainly was struck with the great want of success which followed the operation of lithotomy in the hospitals of Paris, and I attribute it to the faulty means employed. I think the result would have been very different if the lateral method of F. Jaques, the lithotome caché of F. Come, or the *high operation* had been practised for the extraction of the calculi." (p. 82.)

Any reference to loose and uncertain documents for the purpose of establishing the average mortality of lithotomy patients in his own country, has been rendered quite unnecessary by the record of 356 cases occurring in Paris and its environs, furnished by M. Dupuytren in his posthumous work on the bilateral section. Of these 61 died, presenting an average mortality of rather more than one in six. In our review of M. Dupuytren's work, (*vide* Br. and For. Med. Rev., vol. II.) we compared the average mortality in France and England; the summing up was greatly in favour of our own surgeons, since the mean ratio of several series of cases recorded at different institutions was one death in eight operations. We then expressed our conviction that the greater success was mainly dependent on the better method of performing the operation in this country, by which many of those casualties which M. Souberbielle so strongly insists upon as incident to the lateral section are avoided and overcome. We must repeat our former assertion that the French lithotomists seem yet unacquainted with the principle which guides the

English surgeon in opening the bladder; at all events they neither follow it in their practice nor express any clear opinion of it in their works.

The principle and very essence of the lateral section, a principle which involves the subsequent safety of the patient, consists in opening the bladder without entering the cavity of the pelvis, thus forming a direct communication between the interior of the bladder and the external perineal wound, at the same time leaving intact the fascia which lines the cavity of the pelvis. The natural adaptation of this fascia serves to separate the cavity of the pelvis from the lower pelvic opening and from the outlets of the body. In the operation of lithotomy it should always form a barrier between the track of the surgeon's knife and the sub-peritoneal cellular tissue, which becomes exposed and laid open by its lesion. Such an incision, we hold, can only be accomplished with certainty and precision by the gorget or more especially by the knife; we believe its accuracy cannot be insured by the lithotome caché, an instrument almost universally adopted by the French operators. Hence the numerous mishaps and untoward events which have induced M. Souberbielle to abandon the lateral operation and adopt another method, which he considers as less dangerous, but which, if we are to judge from the results, has not been attended with a more successful issue. Our author, in comparing the lateral with the high operation, has of course recorded the risks and dangers which he has experienced and witnessed in the performance of the former. We as English reviewers must claim the privilege of debating the vexed question according to our own experience; and we have no hesitation in affirming that those casualties which our author cites as every day occurrences, are in the English practice very rarely met with. Thus wounds of the rectum and stercoraceous fistulæ, impotence or incontinence are scarcely recognized and certainly not feared as the consequences of lithotomy. Urinary infiltration into the subperitoneal cellular tissue, a most fatal occurrence, very seldom takes place, because, except in cases of peculiar difficulty, the integrity of the pelvic fascia is carefully preserved. It must be remembered that one out of M. Souberbielle's thirty-nine patients died in consequence of urinary infiltration beneath the peritoneum.

The liability to hemorrhage is certainly greater in the lateral operation; but provided the patient is carefully watched and attended to, it very seldom leads to a fatal result, because it can be controlled by the judicious application of pressure; whereas in the case of fatal hemorrhage which M. Souberbielle relates, no means to compress the bleeding vessels could be devised, and the patient sunk from loss of blood.

Although wounds of the peritoneum are disregarded by our author, we certainly should not like to expose our patient to the risk of laying open the serous cavity of the belly; and although in the fatal case related by M. Souberbielle, and already referred to in this article, no evidences of peritonitis were found, we are by no means assured that the collapse and subsequent vital depression which frequently follows abdominal wounds, might not in this instance have hastened if it did not give rise to the death of the individual. We consider that the lateral section through the perineum offers one great advantage to the patient which is not obtained in the high operation, inasmuch as it opens the bladder in the most depending part, in the situation of its natural outlet towards which all the muscular contractions of the viscus tend. It thus ensures a ready passage for the urine to drain away, and facilitates the expulsion of clots of blood, mucous secretions, or perhaps fragments of friable calculi. A stream of water may be easily passed into the bladder through the lower opening, and thus particles of stone may be got rid of whose expulsion could not have been effected in any other way. It likewise renders unnecessary the nuisance of wearing a catheter, the constant presence and irritation of which must, we imagine, in some cases be almost intolerable to the patient, and totally prevent that calmness and quietude which is so essential to his recovery.

It only remains for us to advert to the last point on which we find ourselves at issue with M. Souberbielle, viz., the frequent occurrence of calculi adherent to, impacted in or firmly retained by, the parietes of the bladder. He states (p. 89), "the *fixity* of stones to the walls of the bladder, under which condition the high operation offers so many advantages, is of more frequent occurrence than is generally supposed." Surely the very circumstance that a state of parts said to be so common should hitherto have escaped the attention and comment it deserved goes far to negative the assertion. What is also somewhat remarkable is that M. Souberbielle himself does not appear to have made this notable discovery until he practised the high operation. Then and not till then he is enabled to tell us that out of thirty-nine cases cut above the pubis, sixteen were either so situated, or else so firmly retained by the parietes of the bladder, as would have rendered their extraction by the perineum a matter of the greatest difficulty, nay, that in six it would have been absolutely impossible. We are aware that a stone has sometimes though very rarely become impacted in a pouch formed by the recession of the mucous lining between the bands of the muscular coat. In such instances, either from the absence of symptoms or the difficulty of striking the calculus with a sound, an operation has seldom been attempted, and the fact has been brought to light after death. But with this exception we hardly know of a single instance in which an operator after having fairly opened the bladder has failed to extract the calculus in consequence of its adherence to the walls of the viscus. We believe that M. Souberbielle has mistaken cause and effect; that he made his difficulties himself by incising the bladder through its muscular walls, thereby causing a sudden and violent contraction of the parietes upon their contents, requiring the free use of his fingers and divers other instruments to extricate the stones from the firm grasp which had seized and retained them. We believe that if he had operated by the perineum and opened the bladder without lesion of its muscular coat this difficulty would not have occurred; that in most of the instances he cites the stones would have fallen within the grasp of his forceps, and would have been extracted with the ordinary facility. In the eleven cases when he operated by the lateral section no such obstruction occurred; moreover they all got well.

Of the fifty cases which form the subject of M. Souberbielle's memoir, eleven underwent the lateral operation and they all speedily recovered. It must, however, be observed that the greater number were children and in a condition in which success might fairly be anticipated. Of the remaining 39, 11 died, or 1 in $3\frac{3}{11}$, in round numbers about 4 in 13.

This certainly presents a fearful rate of mortality; but on referring to the table we shall find that many of the cases were highly unfavorable, and on that account, according to our author's views, more particularly adapted for the high section. Many of the patients were advanced in years, in some the calculi were of large size, whilst others had been previously subjected to the manipulations of unsuccessful lithotrity. The result therefore is not to be taken as a criterion of the merits of the operation or as a measure of the success which may be anticipated in the ordinary average of cases.

Although it has been our misfortune to differ materially from M. Souberbielle in many important points, it is far from our intention to represent the high operation as wholly inadmissible or to stigmatise it as mischievous. We should be inclined to give it the preference in some of those cases where the size of the stone and the enlarged and diseased condition of the prostate would lead us to anticipate great difficulty in the extraction with its almost necessary consequence, contusion of the soft parts around the wound and laceration of the cellular tissue and fascia surrounding the prostate and neck of the bladder. Calculus may also be complicated with certain other conditions and varieties under which the bladder may be incised with advantage above the pubes. Our object in this article has been to investigate fairly and impartially the facts and arguments by which M. Souberbielle seeks to justify his preference for the high operation, and we feel quite satisfied in recording our opinion that its general substitution for the ordinary method would not be attended with benefit to the suffering patients; that in the large majority of instances the high operation fails to obviate those evils which indeed are inseparable from unfavorable cases, whilst, on the other hand, it not unfrequently tends to increase the difficulties and give rise to new dangers from which the lateral section is exempt.

---

